# Malaria epidemiology and stratification of incidence in the malaria elimination setting in Harari Region, Eastern Ethiopia

**DOI:** 10.1186/s40249-020-00773-5

**Published:** 2020-11-22

**Authors:** Endashaw Esayas, Asefa Tufa, Fekadu Massebo, Abdulhamid Ahemed, Ibssa Ibrahim, Dereje Dillu, Eyuel Asemahegn Bogale, Solomon Yared, Kebede Deribe

**Affiliations:** 1 Harari Regional Health Bureau, Malaria Control and Elimination Program, Harar, Ethiopia; 2 Malaria and Neglected Tropical Diseases Research Directorate, Armauer Hansen Research Institute, Addis Ababa, Ethiopia; 3 Department of Biology, Arba Minch University, Arba Minch, Ethiopia; 4 Federal Ministry of Health, Addis Ababa, Ethiopia; 5 Jigjiga University, Jigjiga, Ethiopia; 6 Global Health and Infection Department, Brighton and Sussex Medical School, Brighton, 19PX BN UK; 7 School of Public Health, College of Health Sciences, Addis Ababa University, Addis Ababa, Ethiopia

**Keywords:** Malaria elimination, Epidemiology, Incidence, Harari region, Ethiopia, Interventions, Sub-district, Stratification

## Abstract

**Background:**

Ethiopia has shown notable progress in reducing the burden of malaria over the past two decades. Because of this progress, the country has shifted efforts from control to elimination of malaria. This study was conducted to analyse the malaria epidemiology and stratification of incidence in the malaria elimination setting in eastern Ethiopia.

**Methods:**

A retrospective study was conducted to analyse the epidemiology of malaria by reviewing the district health office data from 2013 to 2019 in Harari Region. In addition, three years of sub-district level malaria data were used to stratify the malaria transmission intensity. Malaria interventions (Long-lasting insecticidal nets [LLIN] and indoor residual spraying [IRS]) employed were reviewed to analyse the intervention coverage at the Regional level. Descriptive statistics were used to show the malaria transmission in terms of years, season and species of the malaria parasite. Incidence rate per 1000 population and death rate per 1 000 000 population at risk were computed using the total population of each year.

**Results:**

In the Harari Region, malaria incidence showed a more pronounced declining trend from 2017 to 2019. *Plasmodium falciparum*, *P. vivax* and mixed infections accounted for 69.2%, 30.6% and 0.2% of the cases, respectively. There was an increment in malaria intervention coverage and improved malaria diagnosis. In the year 2019 the coverage of LLIN and IRS in the Region were 93.4% and 85.1% respectively. The annual malaria incidence rate dropped from 42.9 cases per 1000 population in 2013 to 6.7 cases per 1000 population in 2019. Malaria-related deaths decreased from 4.7 deaths per 1 000 000 people annually in 2013 to zero, and there have been no deaths reported since 2015. The malaria risk appears to be heterogeneous and varies between districts. A higher number of malaria cases were recorded in Erer and Jenella districts, which constitute 62% of the cases in the Region. According to the sub-district level malaria stratification, there was shrinkage in the malaria transmission map and about 70% of the sub-districts have achieved elimination targets.

**Conclusions:**

In the Harari Region, malaria morbidity and mortality have been significantly declined. Thus, if this achievement is sustained and scaling-up of the existing malaria prevention and control strategies by focusing on those populations living in the higher malaria transmission districts and sub-districts, planning of malaria elimination from the study area might be feasible.

## Background

Malaria poses threats to the public health of the world, with a particular burden of disease in sub-Saharan Africa [1]. Globally, in 2018 there were an estimated 228 million new malaria cases and, of these, about 405 000 malaria-related deaths. Deaths are most common in children under the age of five years in sub-Saharan Africa. The World Health Organization (WHO) African Region bearing the highest burden of malaria cases (93%) and deaths (94%) [2].

In Ethiopia, the burden of malaria continues to cause a substantial number of morbidity and mortality which accounts for most outpatient visits. Malaria has been one of the main causes of hospitalization and deaths in the country [3, 4]. About 60% of the population are living in the malaria risky areas, mainly areas that lie below 2000 m above sea level [4, 5]. However, several pockets with micro-epidemiological conditions supporting malaria transmission occur in areas above this altitude [6, 7]. *Plasmodium falciparum* and *P. vivax* are the dominant parasites responsible for the majority of malaria cases in Ethiopia. The contribution of other *Plasmodium* species in the country is negligible [5, 8].

Since 2000 there has been a substantial increase in investment to support malaria interventions in Africa, including Ethiopia. Due to this commitment over the past two decades, a significant reduction of malaria has been registered in Ethiopia. This improvement could be attributed to improved coverage of key antimalarial interventions, including, distribution of long-lasting insecticidal nets (LLIN) through mass campaigns targeting the entire population at risk, indoor residual spraying (IRS) in designated epidemic-prone areas, and expanded diagnostic testing and effective antimalarial treatment to people at risk [5, 8–10]. Based on this progress, Ethiopia has recently planned to achieve nationwide malaria elimination by 2030 by starting sub-national elimination in districts with low malaria transmission [8].

Evaluation of changes in malaria epidemiology and malaria control interventions are used for public health surveillance and monitoring, forecasting, program evaluation, policy analysis and investigation of potentially causal relationships between risk factors and outcomes [3, 11, 12]. Moreover, identifying locations and populations with a higher risk of malaria would help appropriate planning and implementation of targeted intervention strategies against the disease to further reduce the risk of infection [13–15]. Thus, analyzing transmission intensity and the interventions employed each year is important for the expansion of intervention strategies or to design new ones to tackle the disease. Harari Region was selected by the National Malaria Elimination Program for the possible malaria elimination and as such, a malaria elimination strategy was launched and is under implementation since 2017. However, the comprehensive evaluation of changes in regional malaria cases and progress made in malaria control interventions and the malaria epidemiology are not studied in the Region. Therefore, this study was aimed at analyzing the epidemiology of malaria and the coverage of interventions employed in the Harari Region. Furthermore, the present study stratifies the malaria transmission intensity at the sub-district levels, which may contribute to the evidence-based decision for malaria control and elimination strategies.

## Methods

### Study area description

**Fig. 1:**
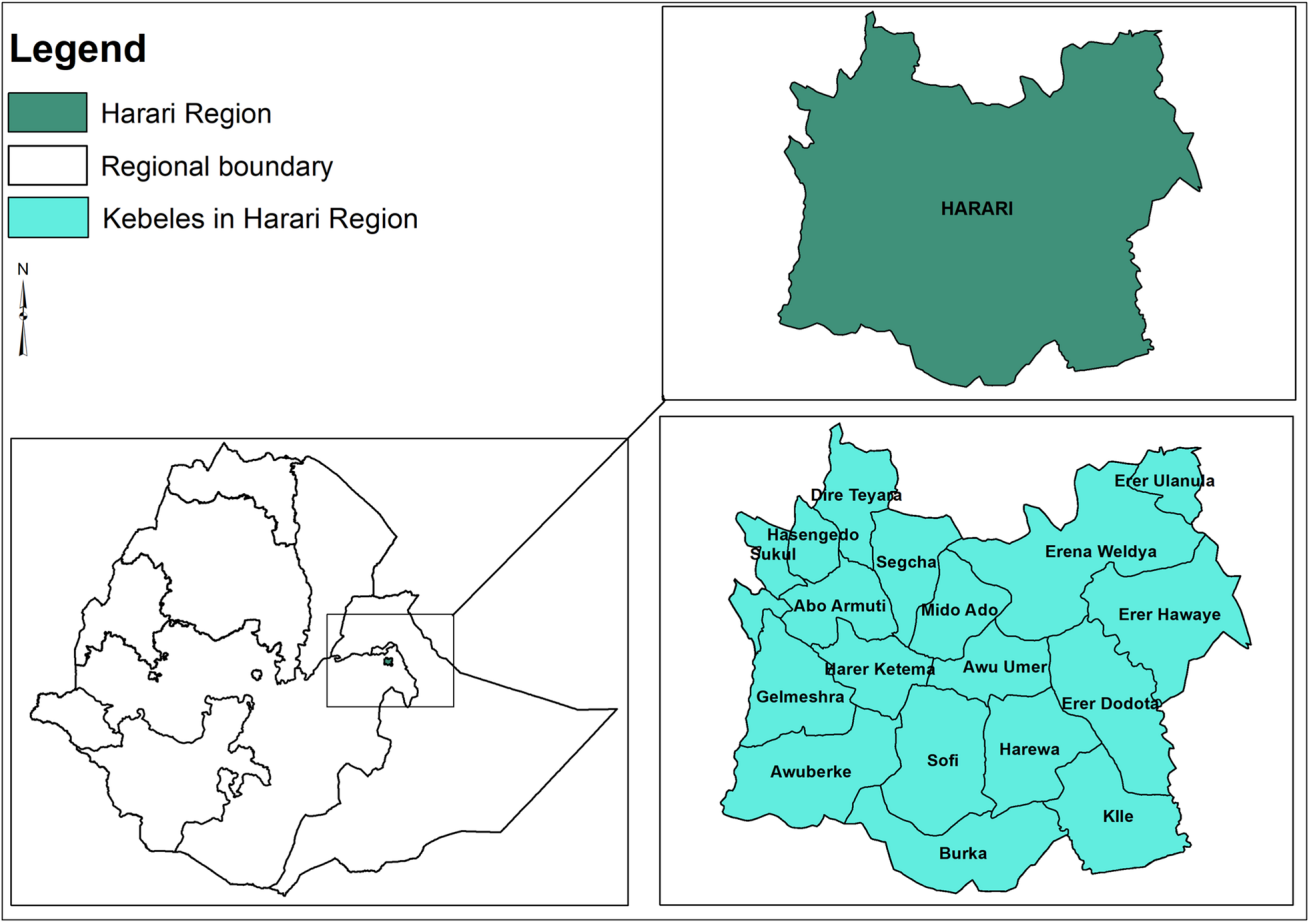
Location of Harari Region in the map of Ethiopia and study sub-districts (*kebeles*)

This study was carried out in Harari Regional State, one of the Regional States of Federal Democratic Republic of Ethiopia, located in the eastern part of the country 515 km from the national capital Addis Ababa. The region is surrounded by Oromia Regional State (Fig. 1). The Region covers a total area of 342.2 square km with an estimated total population of 270 000. The Region is located within 9° 11′ 49″–9° 24′42″ N Latitude and 42° 03′ 30″–42° 16′ 24″ E Longitude and the altitude of the Region ranges between 1552 and 1957 m above sea level. The mean annual temperature of the Region ranges from 18 to 21 °C and the mean annual rainfall ranges from 687 to 775 mm. The Region is divided into three rural districts (Dire Teyara, Sofi and Erer districts) and 6 urban districts within Harar Town Administration (Abadir, Aboker, Amir Nur, Jenella, Hakim and Shenkor districts). These districts are administratively further sub-divided into 36 sub-districts (*kebeles*). Since 2016, the potential primary health service coverage in the Region is estimated to be close to 100%. In Harari Regional State there are six hospitals (two public, two military, a private and a non-governmental), one Regional laboratory and research centre, eight health centres, 32 health posts and 40 private clinics [16]. In addition to the regional people, the health facilities in the region are giving services for the eastern part of Ethiopia. For instance, the Regional laboratory is serving as a testing centre for early infant diagnosis for health facilities of the eastern Hararge zone of Oromia Region, Dire Dawa City Administration and Somali Region.

### Study design

A retrospective study was conducted to analyse the epidemiology of malaria by reviewing the seven years (from January 2013 to December 2019) total malaria data of the district health offices. In addition, three years (from January 2017 to December 2019) malaria data were also used to stratify the malaria transmission intensity at the sub-district level. Furthermore, malaria interventions (LLIN and IRS) employed from 2013 to 2019 were reviewed to analyse the intervention coverage at Regional level.

### Malaria case and data management in the Harari Region

In the Harari Region, malaria is diagnosed clinically and using parasitological tests. The clinical diagnosis is primarily fever or a history of fever (a febrile illness) with or without other symptoms that are suspected by a health worker to be a malaria infection. Parasitological diagnosis, on the other hand, is confirmation of malaria cases by using a diagnostic test (light microscopy [gold standard] or rapid diagnostic tests [RDTs]) [10, 17]. Malaria services such as malaria testing, treatments, distribution of nets are rendered in all health facilities (health posts, health centres and hospitals) in the Region. In health posts, patients are treated clinically or using RDTs. The health posts report malaria case data monthly to their nearest health centre health management information system office (HMIS). In the Region, each health centre has on average four health posts. In addition, confirmed malaria cases are reported monthly from laboratory units to the corresponding health centre and hospital HMIS. The Region strictly follows the standard operating procedures (SOPs) in all phases of quality control. In the Region, peripheral smear examination of a well-prepared and well-stained blood film is used as the gold standard in confirming the presence of the malaria parasite as per the WHO protocol [17]. All hospitals and health centres in the Region follow a SOP for capillary blood sample collection, smear preparation, staining techniques and blood film examination for malaria parasite detection. Finally, all data for malaria cases are reported monthly from each health centre and hospital to the district HMIS. Data are stored electronically in HMIS of the district by month, year, clinically treated, RDT and microscopically confirmed cases. The present study included all malaria cases data recorded between 2013 and 2019 of the Harari Regional State.

Besides case management being strengthened in all districts, the malaria interventions (LLIN and IRS) were targeted to suit the local epidemiological situations in the Region. LLIN were provided free of charge in all districts of the Region and the distribution campaigns were carried out in 2013, 2016 and 2019. IRS is also implemented in all districts of the Region and in the study period, Bendiocarb and Propoxur were the main insecticides used for IRS. Reports on LLIN and IRS coverage from 2013 to 2019 were obtained from the Harari Regional Health Bureau for the current study.

### Data collection

A format was prepared to collect the secondary data from the districts and Regional databases. With the help of the HMIS expert, individual data, such as total confirmed and clinically treated malaria cases in month and year, types of malaria species and addresses of patients were collected from the recorded sheet. In addition, the reported cases and deaths for all ages and age < 5 years between 2013 and 2019 were reviewed. The incidence rate was expressed as malaria cases/1000 people/year and death rate as deaths/1 000 000 people/year. During data collection, the data were strictly checked for completeness and any data such as addresses of patients, and malaria diagnosis results which were not properly recorded were excluded from the analysis. In this record review, a very small number (only eight individual data) were excluded due to incompleteness of registration with missing information on either the address of the patient or malaria diagnosis result (types of malaria species).

The sub-district level annual parasite incidence (API) were analysed to characterize the sub-district as malaria-free (API = 0), low (0 < API < 5), moderate (5 ≤ API < 100), or high transmission (≥ 100) and to map transmission dynamics [8]. In the Harari Region, malaria records at the district and sub-district level were only available from 2013 and 2017, respectively. Annual district level micro-plans were used to determine API of each sub-district from 2017 to 2019. Data from a total of 36 sub-districts and 9 districts were analysed.

### Statistical analysis

Data were first entered into Excel and then imported into IBM® SPSS® (version 20, IBM Corporation, Armonk, New York, US) for analysis. Descriptive statistics were used to show the trends of malaria transmission in terms of season, years and species of the malaria parasite. Pearson’s Chi-square test was used to describe the associations of variables. Test of significance was estimated assuming α at 0.05 and a *P*-value less than 0.05 was considered significant. The API per 1000 population at risk was computed using the total population of each year as a denominator and confirmed cases of malaria as a numerator. API was calculated based on the current sub-district population, 2007 Ethiopian government census and 2013 projections at district and regional levels assuming constant population growth rate during the study period. Numbers of sub-districts, the total population and percentage composition in different transmission categories (malaria-free, low, moderate and high) were calculated and compared between 2017 and 2019. Percent changes in malaria indicators from 2013 to 2019 were calculated according to the formula: (Value2013 − Value2019)/Value2013 × 100. Malaria positivity rate was calculated as the number of confirmed cases over the total suspected febrile case examined at study sub-districts. Seasonality was determined by the monthly positivity rate of total infections and by species. Species composition was calculated annually. Finally, all maps were produced using ArcGIS Desktop (v 10.5, Environmental Systems Research Institute Inc., Redlands CA, USA). The base map of the global administrative areas was downloaded from the Natural Earth (https://www.naturalearthdata.com/).

## Results

### Annual reported malaria cases in the Harari Region

From 2013 to 2019, a total of 44 882 (46.9%) malaria cases were detected from 95,629 malaria-suspected outpatients diagnosed in the Harari Region with annual mean malaria cases of about 6412. Of these, 41 046 were confirmed malaria cases (annual mean = 5864) while 3836 were reported as clinical cases in the Region. From the confirmed cases of malaria, 33 575 (81.8%) were identified by microscopy, whereas 7471 (18.2%) cases were diagnosed by RDT. Despite malaria cases being reported in all years, there was a fluctuating trend of malaria within the study period. In 2016, the highest number of malaria-suspected patients (*n* = 21 671) were examined and of these 11 605 (53.6%) were confirmed malaria cases whereas the least number of cases (1767 [12.5%]), were reported in 2019 (Table 1).

**Table 1:** Malaria suspected cases examined, parasitological confirmed and clinically identified malaria cases in Harari Region from 2013 to 2019

Year	Malaria suspected cases examined	Total malaria cases (confirmed + clinical)	Confirmed malaria cases				Clinical malaria cases (%)
			Pf *n* (%)	Pv *n* (%)	Mixed *n* (%)	Total *n* (%)	
2013	11 502	10 448 (90.8)	5256 (58)	3780 (41.7)	33 (0.3)	9069 (86.8)	1379 (13.2)
2014	5330	4105 (77.0)	1961 (48.1)	2113 (51.9)	0 (0.0)	4074 (99.2)	31 (0.8)
2015	9536	5981 (62.7)	3896 (69.4)	1704 (30.3)	14 (0.3)	5614 (93.9)	367 (6.1)
2016	21 671	11 605 (53.6)	8352 (78.2)	2302 (21.6)	26 (0.2)	10 680 (92.0)	925 (8.0)
2017	18 814	8712 (46.3)	5825 (76.4)	1792 (23.5)	4 (0.1)	7621 (87.5)	1091 (12.5)
2018	14 658	2264 (15.5)	1518 (68.3)	703 (31.7)	0 (0.0)	2221 (98.1)	43 (1.9)
2019	14 118	1767 (12.5)	1585 (89.7)	182 (10.3)	0 (0.0)	1767 (100)	0 (0.0)
Total	95 629	44 882 (46.9)	28 393 (69.2)	12 576 (30.6)	77 (0.2)	41 046 (91.4)	3836 (8.6)

*Pf* *Plasmodium falciparum; Pv P. vivax*

**Fig. 2:**
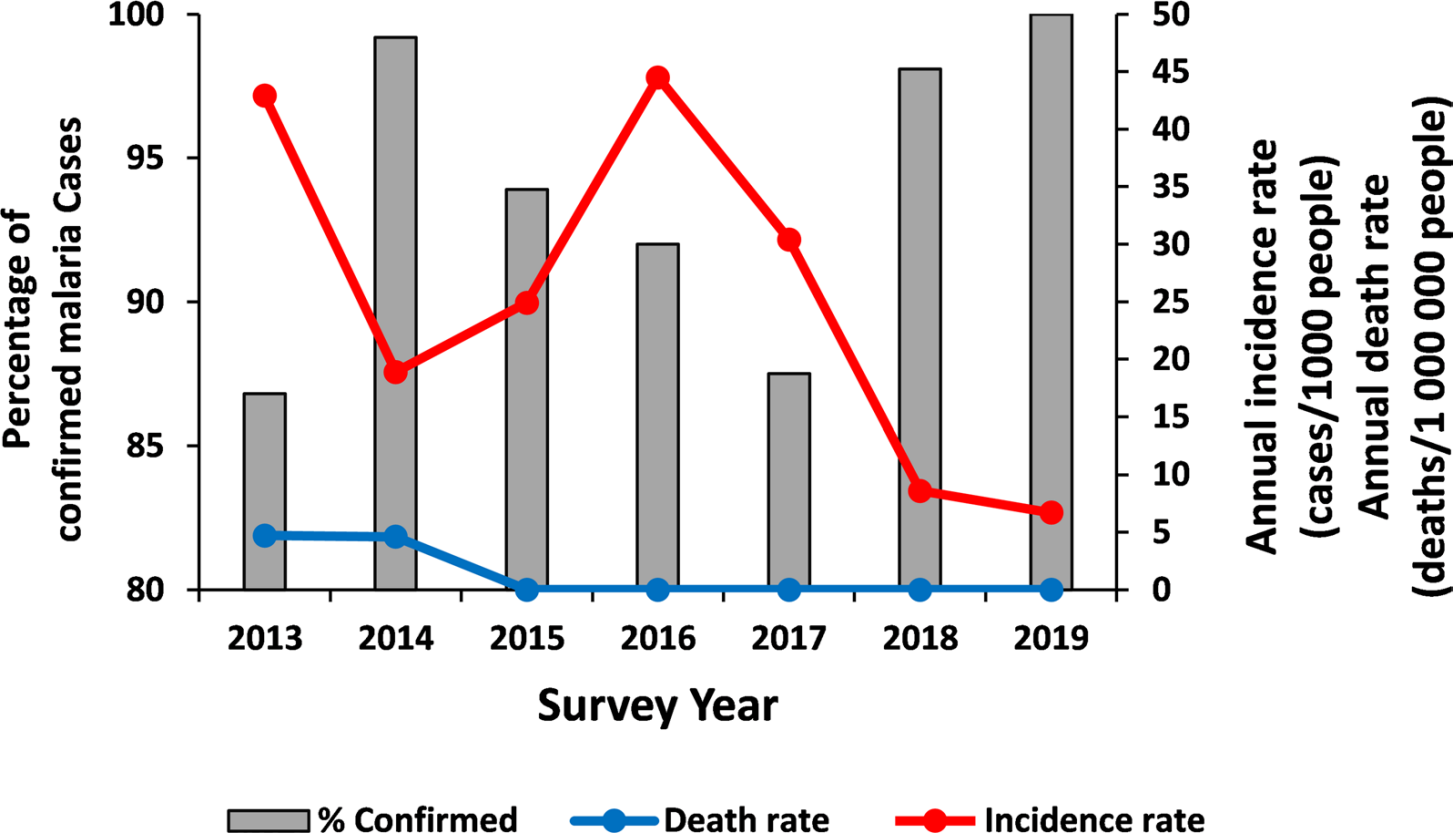
Annual total reported malaria cases, malaria-related deaths and proportion of parasitologically confirmed cases in Harari region from 2013 to 2019

In general, there was a decreasing trend in the number of confirmed malaria cases from 2013 to 2014 which was followed by an increasing trend from 2014 to 2016 (the average annual incidence was 29.9 cases per 1000 people). The malaria incidence showed a pronounced declining trend from 2017 to 2019, where the average annual incidence was 15.2 cases per 1000 people. The incidence of malaria death was 2.2 deaths per 1 000 000 people annually between 2013 and 2016, and there was no malaria-related death report between 2015 and 2019, a drop of 100% (Fig. 2).

**Fig. 3:**
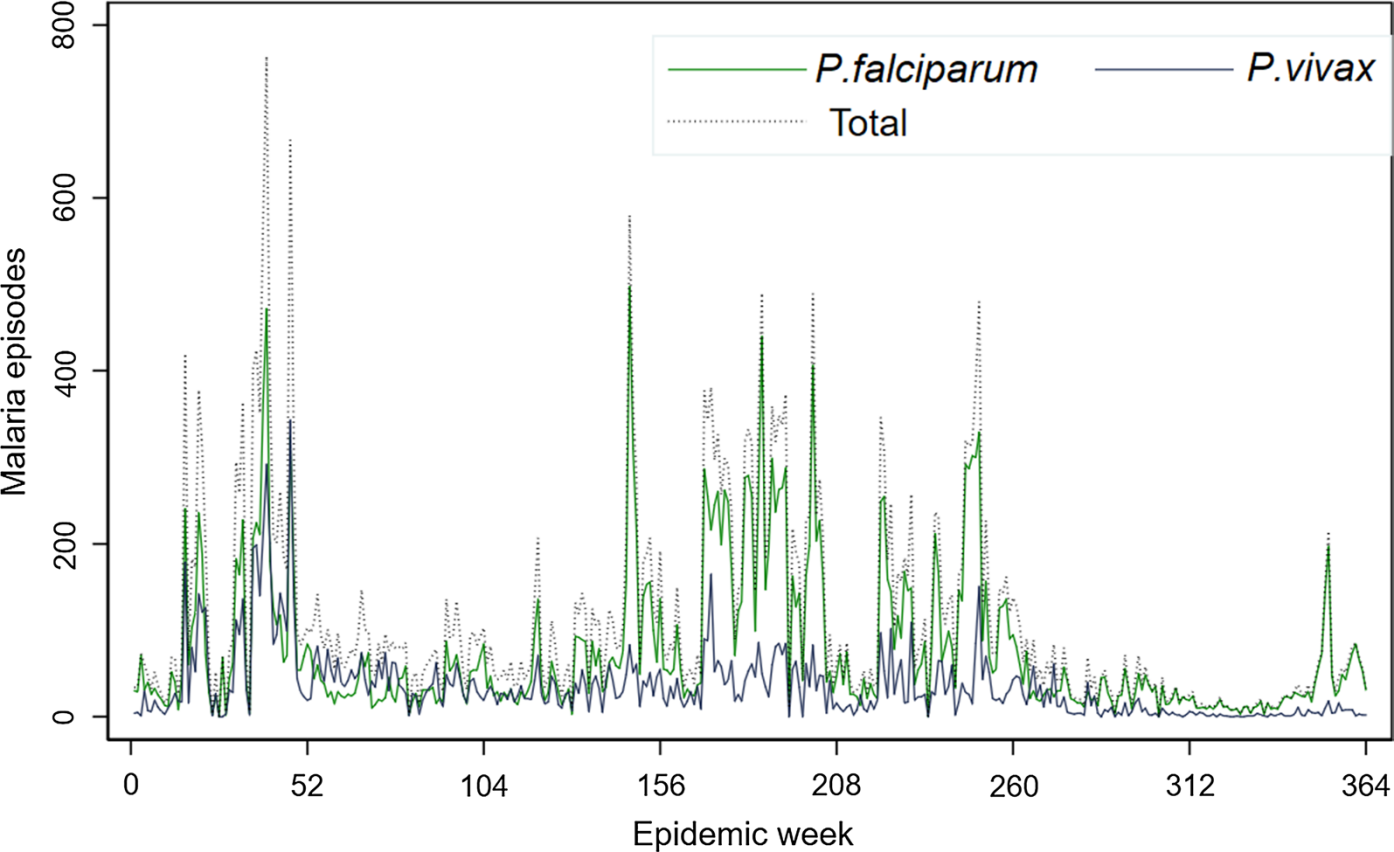
Weekly *Plasmodium* species trend in Harari region from 1^st^ January 2013 to 31st December 2019

With regard to *Plasmodium* species, throughout the reviewed period the numbers of *P. falciparum* cases were dominant over *P. vivax* except the year 2014. Out of the total confirmed malaria cases, *P. falciparum* accounted for 28 393 (69.2%) whereas *P. vivax* 12 576 (30.6%) cases. Mixed *P. falciparum*/*P. vivax* infections were insignificant in the Region which accounted for only 0.2% of total confirmed malaria cases (*n* = 77) (Table 1). The seven-year *plasmodium* species trend in the Harari region indicates *P. vivax* malaria showed the same trend, a drop in the number of cases throughout all years. On the other hand, *P. falciparum* cases consistently increased from 2014 to 2016 and downward trend afterward (Fig. 3).

### Antimalarial interventions coverage in the Harari Region

**Fig. 4:**
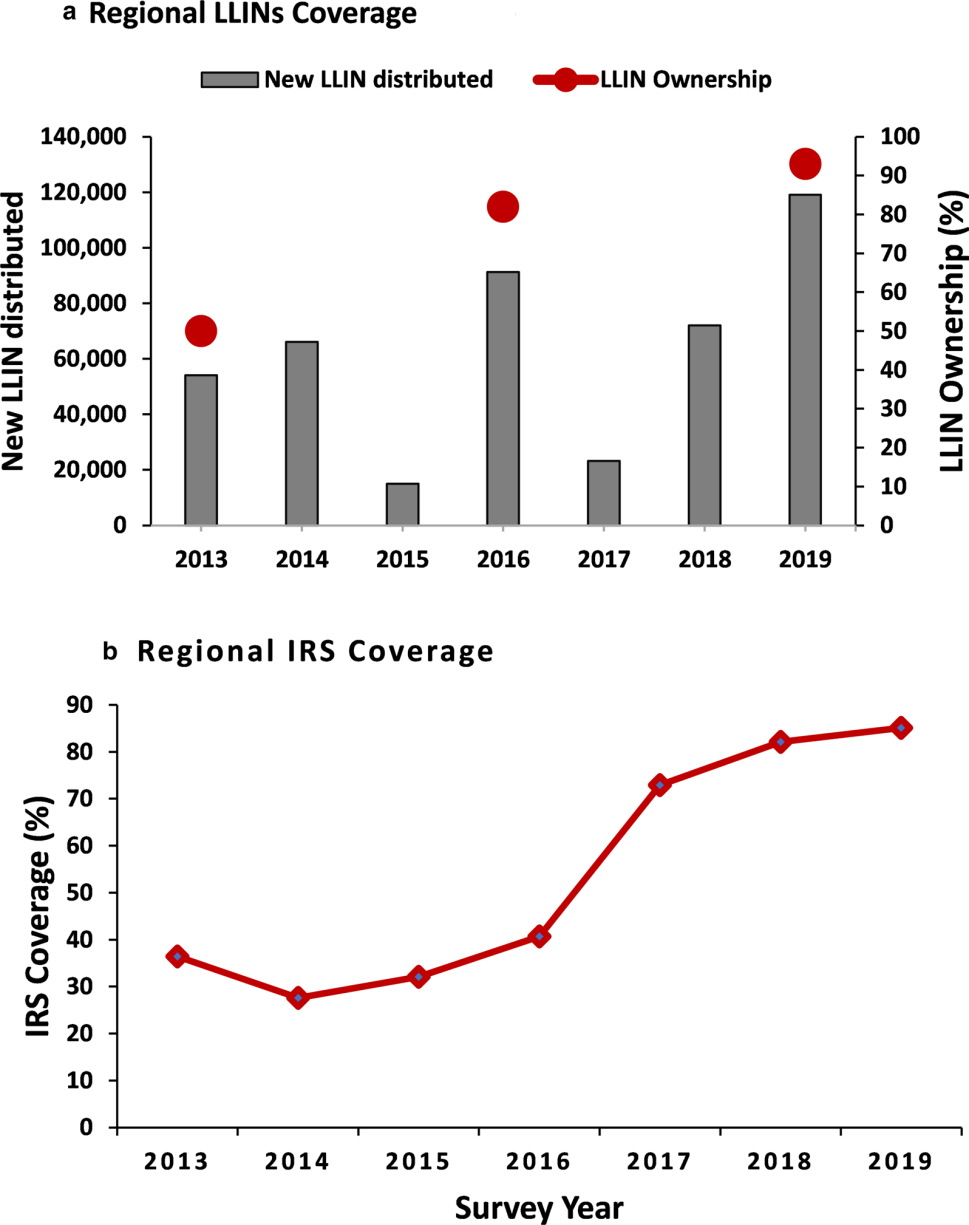
Regional level: **a** Annual total number of new LLIN distributed from 2013 to 2019 and household LLIN ownership (% of households with at least one LLIN) in 2013, 2016 and 2019; **b** Regional IRS coverage (%) from 2013 to 2019. LLIN: Long-lasting insecticidal nets; IRS: Indoor residual spraying

Harari Region has distributed a total of 440 626 LLINs between 2013 and 2019. The proportion of households with at least one LLIN ownership in the Region has increased from 50% in 2013 to 82% in 2016 and 93.4% in 2019 (Fig. 4a). The IRS coverage has also increased from 36.4% in 2013 to 85.1% in 2019 in the Region (Fig. 4b).

### Regional variations in the malaria epidemiology

The annual incidence declined from 42.9 cases per 1000 people in 2013 to 6.7 cases per 1000 people in 2019, a drop of 84.4%. Reported malaria cases in children < 5 years decreased by 93.9%, declining from a total of 1274 in 2013 to 78 in 2019. The overall malaria-related admissions and deaths showed a significant decrease between 2013 and 2019 that malaria-related admission as well as death report were dropped by 100%. The case confirmation rate by microscopy and/or RDT increased from 86.8% in 2013 to 100% in 2019 (Table 2).

**Table 2:** Variations in malaria epidemiological indicators in Harari Region from 2013 to 2019

Year	2013	2019	Percent change (%)
Total reported cases	10 448	1767	− 83.1
Confirmed	9069	1767	− 80.5
(% confirmed)	86.8	100	15.2
Incidence rate (cases/1000 population)	42.9	6.7	− 84.4
*P. falciparum* proportion (%)	58	89.7	54.7
Age			
< 5 years	1274	78	− 93.9
(% of total)	12.2	4.4	− 63.9
≥ 5 years	9174	1,689	− 81.6
Malaria admission			
< 5 years	1	0.0	− 100
(% of total)	16.7	0	− 100
≥ 5 years	5	0	− 100
Malaria deaths			
< 5 years	0	0	0
(% of total)	0.0	0.0	0.0
≥ 5 years	1	0	− 100

### Malaria cases variation in between districts of Harari Region

Regardless of the apparent variation of malaria trends in the study area, malaria cases occurred in almost every district of the Region. Based on year by year reported malaria cases, the highest cases were reported from the Jenella and Erer districts. These two districts experienced 62.4% (28 001/44 882) of the total malaria cases in the Region. Conversely, the least malaria cases were tested and detected in Abadir and Shenkor districts (Table 3).

**Table 3:** Malaria cases distribution within districts of Harari Region from 2013 to 2019

Districts	Total malaria cases							Total Cases *n* (%)
	2013	2014	2015	2016	2017	2018	2019	
Abadir	39	0	6	8	0	0	0	53 (0.1)
Aboker	32	191	36	86	80	19	16	460 (1.0)
Amir Nur	333	234	1552	3352	1790	220	164	7645 (17.0)
Dire Teyara	108	147	281	227	159	57	32	1011 (2.3)
Erer	3831	1172	1161	1303	1565	314	127	9473 (21.1)
Hakim	584	360	590	1238	1039	395	658	4864 (10.8)
Jenella	5179	1910	2164	4751	3084	1049	391	18,528 (41.3)
Shenkor	0	4	0	3	0	54	16	77 (0.2)
Sofi	342	87	191	637	995	156	363	2771 (6.2)
Total	10,448	4105	5981	11 605	8712	2264	1767	44,882 (100)

### Sub-district level stratification of malaria incidence

**Fig. 5:**
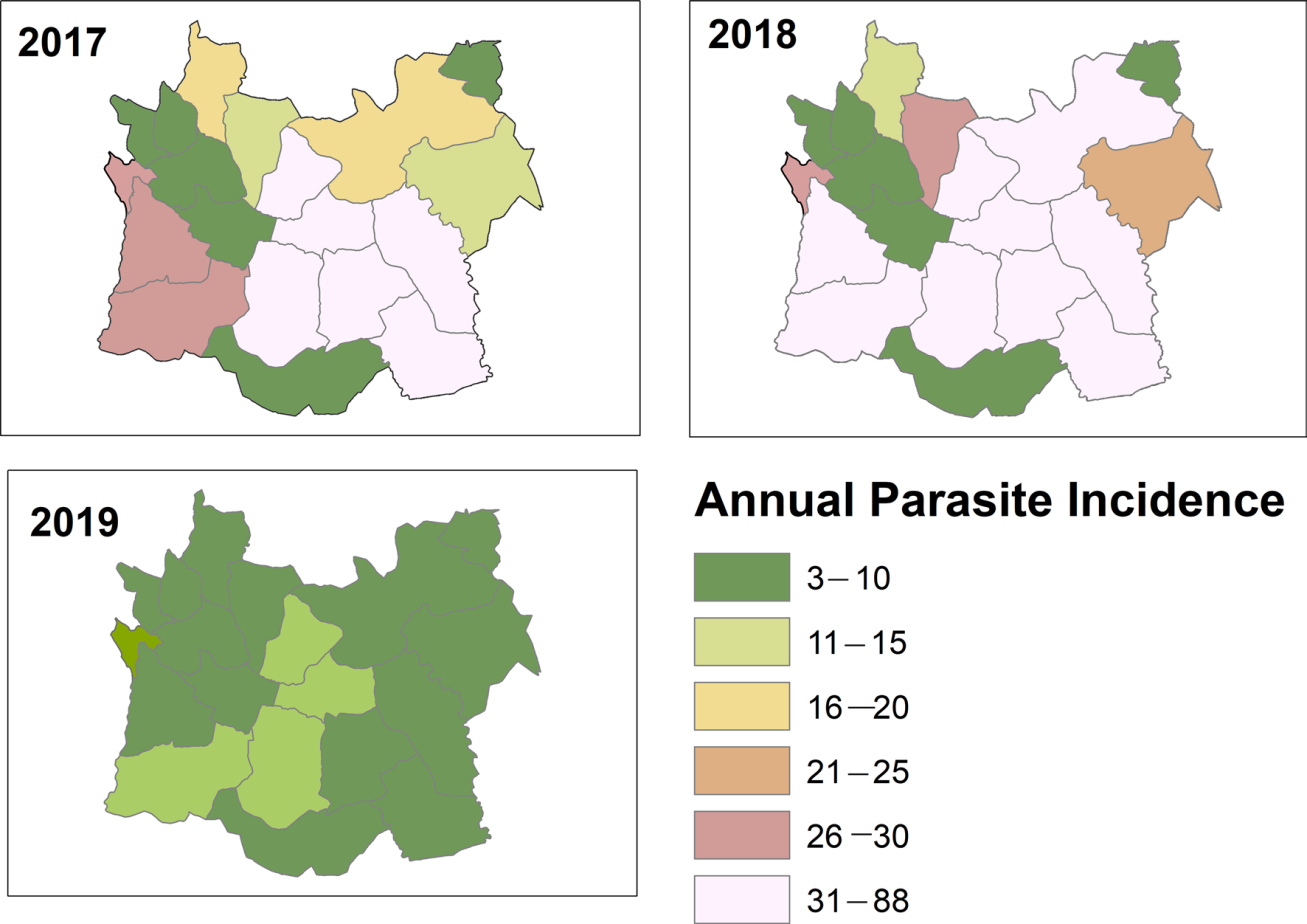
Sub-districts level stratification of malaria incidence from 2017 to 2019, Harari region, Ethiopia

Based on the API per 1000 population, one of the major determinates of malaria transmission which is used for stratification; the sub-districts of the Harari Region stratified into three broad strata of malaria. They are malaria-free (API = 0), low-(0 < API < 5) and moderate-transmission strata (5 ≤ API < 100). Between 2017 and 2019 there were no sub-district with high malaria transmission strata in the Region (Fig. 5).

In the Harari Region, according to the epidemiological data of the region’s malaria stratification, 8.3% of sub-districts reported no malaria and the majority of sub-districts (61.1%) reported fewer than five cases per thousand population. Between 2017 and 2019, the number of moderate transmission sub-districts dropped by 45% (from 55.6% to 30.6%) whereas low transmission districts increased by 37.5% (from 44.4% in 2017 to 61.1% in 2019). The proportion of the population in moderate risk areas decreased from 56.9% in 2017 to 26.8% in 2019, a drop of 52.9% while the proportion in low-risk areas increased by 51% (from 43.1% to 65.1%) (Table 4).

**Table 4:** Variations in the distribution of malaria transmission intensity and affected populations for defined categories of malaria strata at sub-district level in Harari Region from 2017 to 2019

Malaria Strata	API	Number of sub-districts				Percent changes	Total population				Percent changes
		2017	%	2019	%		2017	%	2019	%	
Free	0	0	0	3	8.3	8.3	0	0	21 230	8.1	8.1
Low	0 < API < 5	16	44.4	22	61.1	37.5	108 231	43.1	171 745	65.1	51
Moderate	5 ≤ API < 100	20	55. 6	11	30.6	−45	142 729	56. 9	70 681	26.8	−52.9
High	≥ 100	0	0.0	0	0.0	0.0	0	0.0	0	0.0	0
Total		36		36			250 960		263 656		

API: Annual parasite incidence

**Fig. 6:**
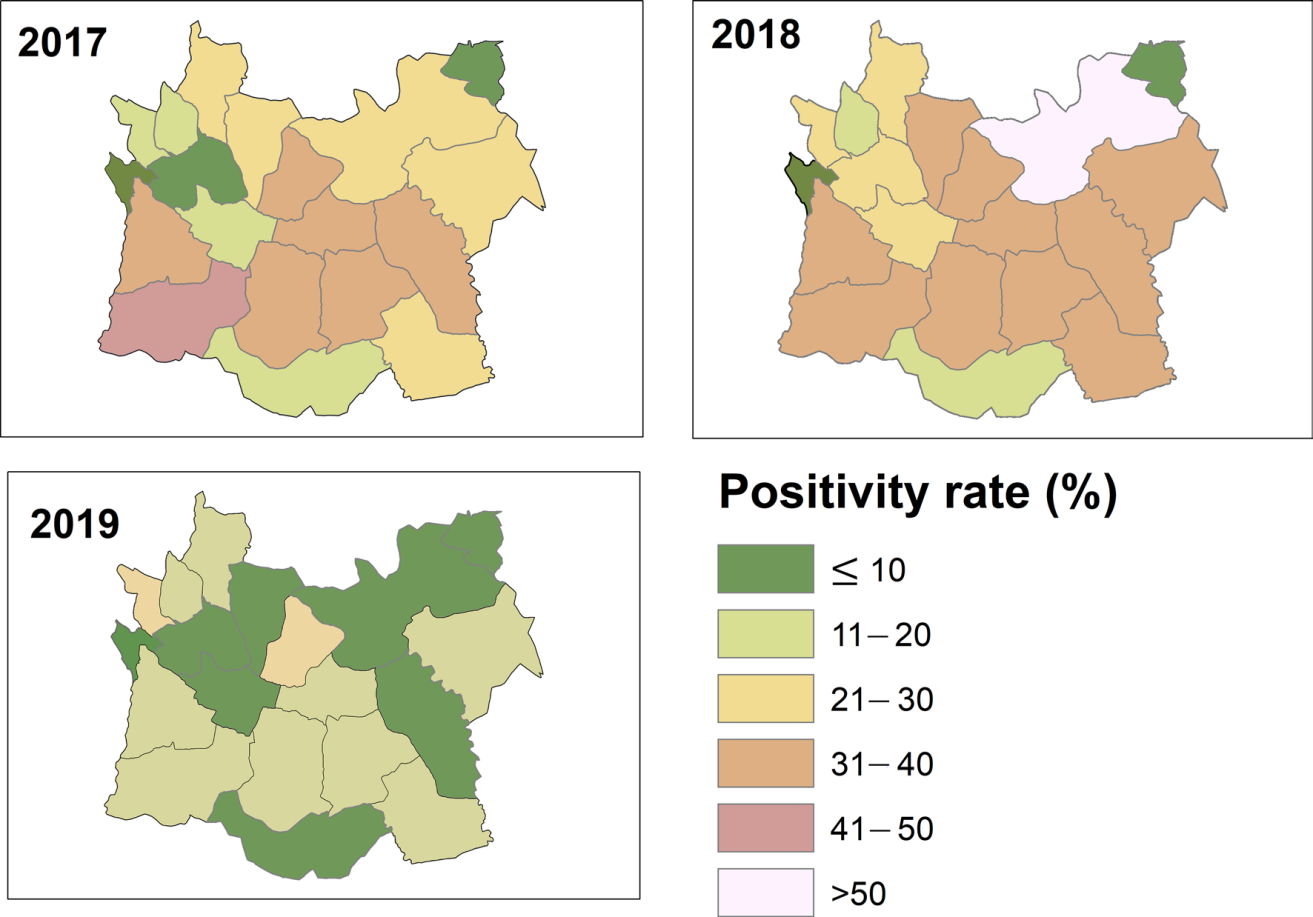
Sub-district level malaria positivity rate from 2017 to 2019, Harari Region, Ethiopia

Based on the malaria positivity rate, one of the major indicators of the malaria case burden in the Harari Region is that the positivity rate has been reduced from 21.9% in 2017 to 10.6% in 2019. Although some sub-districts were below 5%, the malaria test positivity rate is still greater than 10% in some of the elimination sub-districts in the Region (Fig. 6).

### Seasonal variation of malaria cases in the Harari Region

**Fig. 7:**
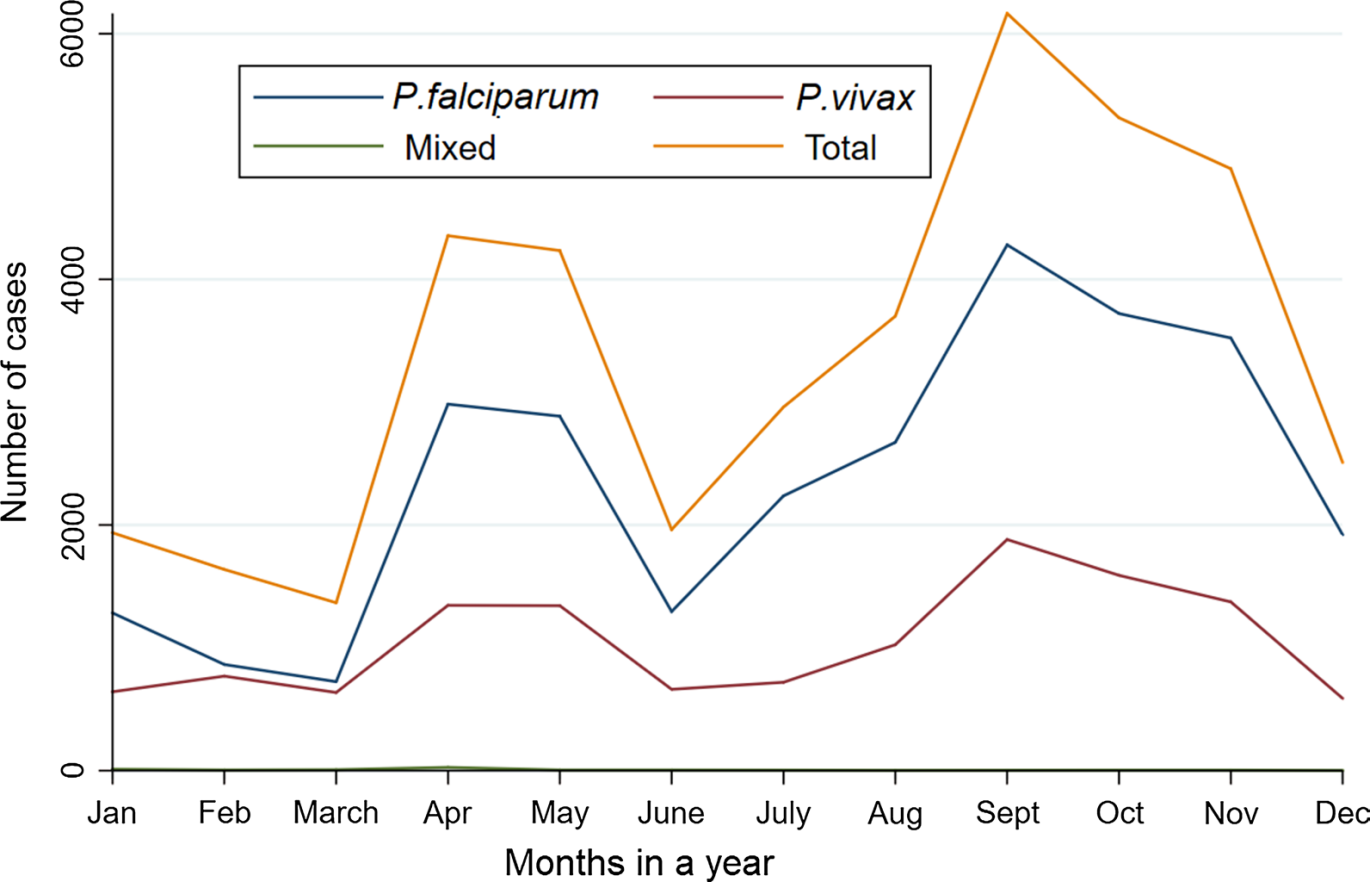
Monthly distribution of *Plasmodium* species and total confirmed malaria cases in Harari Region, from January 2013 to December 2019

In the present study, despite the apparent fluctuation of malaria trends, cases occurred in almost every month of the year in the region. The highest peak of malaria cases in almost all years was observed from September to November. Correspondingly, there was a smaller peak of malaria cases from April to May. The minimum number of malaria cases were observed from December to February. At the species level, the higher number of *P. falciparum* and *P. vivax* cases were diagnosed and identified from September to November whereas the lower number of the malaria parasites were observed from December to February (Fig. 7).

## Discussion

The present study demonstrated that, in the Harari Region, malaria transmission intensity has been significantly reduced and there was a clear decrease in malaria incidence rate between 2013 and 2019. The study elucidates the variation in malaria epidemiology, the annual trends and the seasonal variation of malaria cases and *Plasmodium* species in the Harari Region. In addition, it also shows malaria incidence stratification and transmission patterns at the sub-district level and variation of cases between districts. The current study confirms that malaria risk appears to be heterogeneous and varies greatly between districts. Based on the seven years retrospective data, malaria cases were higher in Erer and Jenella districts and about 62% of the cases were from these two districts. Moreover, according to the sub-district level malaria stratification, there was a shrinkage in the malaria transmission map and about 70% of the sub-districts have reached the elimination levels of transmission. The findings from this study may support the designing of the large-scale malaria interventions and resource mobilization for malaria elimination.

The number of malaria cases in the study area showed a year-to-year heterogeneous trend over the period of 2013 to 2019. Higher malaria-suspected patients and confirmed malaria cases in the Region were observed in 2016. This could be due to several factors such as climatic changes and attendant irregularities in rainfall and temperature, and increased agricultural activities such as irrigation in the study area [18]. On the contrary, malaria incidence showed a more pronounced declining trend from 2017 to 2019. This reduction in malaria cases in the Region was associated with the intensification of the current malaria control and preventive activities by stakeholders. This finding is similar to those of a previous study showing the effect of malaria control interventions on reduction of malaria incidence in central [12, 19] and southern Ethiopia [13]. In the Harari Region, there was high LLIN (as well as LLIN ownership) and IRS coverage which would be expected to decrease malaria transmission further. This result is far higher than the nation-wide average antimalarial intervention coverage of the country [2]. However, there is still a need to improve the utilization rate of LLIN in the Region so that all at-risk populations can be adequately protected. In general, studies from the different regions of Ethiopia [19–21] have shown that there was a decreasing trend of malaria cases in the country. Despite these declining trends, there is still high malaria case incidence, and low LLIN ownership rate and IRS coverage in some malarious areas of the country [12]. Therefore, as shown in the Harari Region, there is plenty of opportunity to strengthen the current malaria control interventions in these malarious areas which could anticipate significant contribution on the further reduction of the current malaria transmission status of the country.

In the study area, *P. falciparum* was the dominant malaria parasite species followed by *P. vivax*. This finding is in agreement with other recent study reports from different parts of Ethiopia [20, 21]. The finding was also in line with the national profile of malaria parasite distribution in Ethiopia which indicates that *P. falciparum* and *P. vivax* are the two dominant malaria parasites, distributed all over the country [5, 22]. The proportion of *P. falciparum* malaria remained nearly unchanged from 2013 to 2019 indicating further efforts are needed to suppress malaria transmission. Nonetheless, in the Harari Region, the proportion of clinical cases (presumptive cases lacking parasitological confirmation) based on symptoms alone has declined greatly in the last seven years and dropped to zero in 2019. This was a result of an improved malaria diagnosis (the malaria test-treat policy). Moreover, the Region has relatively high health services coverage that patients with severe complications of malaria might seek health services particularly in the hospitals in Harari Region from neighbouring Regions (Somali and Oromia).

In the Harari Region, based on the reported malaria cases, the highest number of cases were reported from the Erer and Jenella districts. These districts bore 62% of the total malaria cases in the Region. These differences in malaria cases might be due to differences in climate, topography and malaria diagnostic techniques used. In Erer district there is a seasonal Erer River that originates in the highlands and crosses the district. The River has a high potential for irrigation and the farmers in the district use the water for irrigation along with the coast. This seasonal river may create some puddles around the shore and irrigated villages could be the potential breeding sites for *Anopheles* mosquitoes. High larvae and adult vector and a higher prevalence of malaria were registered in the irrigated village, this could contribute to malaria transmission in the area [15, 23]. The Jenella district is one of the oldest known malaria diagnostic centre in the Region that most of the febrile cases from the Region comes to the Jenella Health centre to be tested for malaria cases which may increase the cases in the district. Therefore, the number of malaria cases reported from the district might not necessarily reflect the number of cases in the district. Clarifying the heterogeneous nature of malaria transmission at the district level in the Region is important in developing dynamic and area-specific risk maps to identify locations and populations at highest risk for appropriate planning, implementation of targeted and epidemiologically sound preventive and control measures against the disease [13, 15]. Furthermore, there is also frequent human movement from Region to Region particularly from neighbouring malaria endemic areas of the Oromia and Somali Regions. This may contribute to the increment of imported malaria cases in these districts that may challenge malaria elimination from the Region.

The sub-districts level stratification of malaria risk based on the three-year annual parasite incidence (API) per 1000 population showed that through the period of 2017–2019 there was an expansion of malaria-free and low transmission stratum. The number of sub-districts reclassified from moderate stratum to low was kept increasing from 2017 to 2019. About 70% of the sub-districts have reached the WHO standard for “elimination” levels of transmission. Furthermore, 8.3% of the sub-districts reported no malaria and there were no high malaria strata in the Region. This may be attributed to the impact of the ongoing malaria control intervention coverage and increased community awareness and participation which updated the Region malaria map. This is in line with the current national malaria picture that demonstrates further shrinking of malaria in the Region [5].

In the Harari Region, seven-year data revealed that the high malaria cases almost all years were recorded from September to November, followed by April to May. These two seasons are the major and minor malaria transmission periods in Ethiopia, respectively. The major malaria transmission period follows the heavy rains from June to August which creates a suitable environment for the breading of *Anopheles* mosquitoes. The minor transmission season occurs between April and May following the small shower of rains from February to March. These peak malaria seasons in the study area are similar to other studies from different parts of the country [20, 21]. This result may suggest that malaria control interventions, such as IRS, in the study area should be designed based on the malaria transmission patterns.

This analysis used the malaria data and interventions coverage from a seven year period at the Regional level and three year period of data at sub-district level. Nonetheless, there are some limitations to the analysis, for API-based stratification of the malaria cases this paper cannot find the sub-district level shapefile of Harar Town. However, the API of these sub-districts was < 1, therefore the current analysis aggregated all cases reported from these sub-districts as malaria cases of Harar Town and for further clarification, it was presented in a table format.

## Conclusions

In conclusion, after the introduction of current malaria control and elimination strategies, malaria cases have shown a significant decline in the Harari Region. Malaria transmission is ubiquitous in the Region, heterogeneous in the distribution and seasonal in the pattern of the number of cases. Over a period of seven years, the burden of malaria has significantly decreased. If this achievement is sustained, the elimination of the disease from the study area may be feasible. However, there is an apparent variation of malaria incidence between districts and sub-districts. Therefore, there is a need for intensifying the existing malaria prevention and control strategies in the Region by focusing on those populations living in the higher malaria transmission districts and sub-districts.

## Data Availability

Data supporting the conclusions and outcomes of this article are included in the article. The raw datasets presented and analyzed in this study are available upon request from the corresponding author.

## Abbreviations

API: Annual parasite incidence
HMIS: Health management information system
IRS: Indoor residual spraying
LLINs: Long-lasting insecticidal nets
SOPs: Standard operating procedures
WHO: World Health Organization
